# Economic Burden of Reported Lyme Disease in High-Incidence Areas, United States, 2014–2016

**DOI:** 10.3201/eid2806.211335

**Published:** 2022-06

**Authors:** Sarah A. Hook, Seonghye Jeon, Sara A. Niesobecki, AmberJean P. Hansen, James I. Meek, Jenna K.H. Bjork, Franny M. Dorr, Heather J. Rutz, Katherine A. Feldman, Jennifer L. White, P. Bryon Backenson, Manjunath B. Shankar, Martin I. Meltzer, Alison F. Hinckley

**Affiliations:** Centers for Disease Control and Prevention, Fort Collins, Colorado, USA (S.A. Hook, A.F. Hinckley);; Centers for Disease Control and Prevention, Atlanta, Georgia, USA (S. Jeon, M.B. Shankar, M.I. Meltzer);; Connecticut Emerging Infections Program, Yale School of Public Health, New Haven, Connecticut, USA (S.A. Niesobecki, A.P. Hansen, J.I. Meek);; Minnesota Department of Health, St. Paul, Minnesota, USA (J.K.H. Bjork, F.M. Dorr);; Maryland Department of Health, Baltimore, Maryland, USA (H.J. Rutz, K.A. Feldman);; New York State Department of Health, Albany, New York, USA (J.L. White, P.B. Backenson)

**Keywords:** Lyme disease, cost of illness, societal cost, current procedural terminology, vector-borne diseases, New York, Connecticut, Maryland, Minnesota, United States, bacteria, Borrelia burgdorferi, Ixodes scapularis, Ixodes pacificus, ticks

## Abstract

Approximately 476,000 cases of Lyme disease are diagnosed in the United States annually, yet comprehensive economic evaluations are lacking. In a prospective study among reported cases in Lyme disease–endemic states, we estimated the total patient cost and total societal cost of the disease. In addition, we evaluated disease and demographic factors associated with total societal cost. Participants had a mean patient cost of ≈$1,200 (median $240) and a mean societal cost of ≈$2,000 (median $700). Patients with confirmed disseminated disease or probable disease had approximately double the societal cost of those with confirmed localized disease. The annual, aggregate cost of diagnosed Lyme disease could be $345–968 million (2016 US dollars) to US society. Our findings emphasize the importance of effective prevention and early diagnosis to reduce illness and associated costs. These results can be used in cost-effectiveness analyses of current and future prevention methods, such as a vaccine.

Lyme disease is a bacterial illness caused primarily by infection with *Borrelia burgdorferi*, transmitted by the bite of infected *Ixodes scapularis* and *I. pacificus* ticks in the United States. Early symptoms can include a rash known as erythema migrans and influenza-like symptoms (*1*). Disseminated infection can cause neurologic, musculoskeletal, and cardiac complications; in rare cases, cardiac involvement can be fatal (*1*–*4*). Most patients will experience a full recovery after antibiotic treatment, although a minority may continue to experience symptoms related to disease sequelae (*1*).

Lyme disease case numbers consistently rank in the top 10 among all nationally notifiable conditions, and it is the most commonly reported vector-borne disease in the United States (*4*,*5*). Annually, >30,000 cases are reported to the Centers for Disease Control and Prevention (*4*), but recent studies have demonstrated that the annual number of diagnosed cases is ≈476,000 (*6*). This figure represents a substantial disease burden, but the total economic cost to US society is unknown (*7*).

Economic evaluations for Lyme disease have limitations (*7*). Most studies report direct medical costs but lack data on nonmedical costs and losses in productivity (*8*–*11*). Several studies were conducted >2 decades ago in a few Maryland counties where Lyme disease was emerging (*9*,*11*,*12*); however, this limited scope prevents generalizability to other areas in which the disease is endemic, and results might not be representative of today’s costs because of changes in disease management and healthcare structures. More recent studies have used diagnosis codes (e.g., International Classification of Diseases, 9th Revision, Clinical Modification) to identify Lyme disease patients from insurance claims databases. However, the low sensitivity and specificity of these codes in identifying actual cases (*13*,*14*) might lead to incorrect estimates of direct medical costs attributable to the disease. The few studies that provide more comprehensive cost estimates of Lyme disease were conducted in Europe under healthcare systems with financing structures different from those of the United States (*15*–*17*). As such, updated estimates of the total societal cost of Lyme disease, including direct and indirect costs, are needed in the United States (*7*).

We aimed to address current research gaps by conducting a prospective cost-of-illness study to estimate the economic burden of reported Lyme disease in high-incidence areas of the United States. The main objectives of this study were to estimate the patient cost and the societal cost per participant. The secondary objective was to evaluate the association of select disease and demographic factors with the societal cost per participant. Results can be used by public health officials and communities to assess the cost-effectiveness of interventions to reduce the incidence of Lyme disease.

## Methods

### Study Design

This study was conducted as part of TickNET, a public health network of researchers who collaborate on tickborne disease research and surveillance (*18*). We conducted a prospective cost-of-illness study to estimate total costs per patient caused by Lyme disease in 4 high-incidence states: Connecticut, Maryland, Minnesota, and New York. We used an incidence-based design, measuring the cost of illness from onset to conclusion (*19*,*20*). We analyzed these costs from the patient perspective (i.e., costs incurred by the patient) and the societal perspective (i.e., costs incurred by the patient, healthcare system, or third-party payer) (*21*,*22*). Cost categories included direct medical costs (clinician visits, procedures, diagnostic testing, therapy, hospitalization, emergency department visits, or other relevant costs); direct nonmedical costs (roundtrip travel costs for healthcare visits and amount paid for assistance with self-care, dependent care, or house or yard maintenance because of Lyme disease); and indirect costs, which are the cost of productivity losses (time taken off work or school because of symptoms or healthcare visits) (Appendix Table 1) (*23*). Henceforth, we will refer to direct medical costs as either patient medical costs (for medical costs borne by the patient) or societal medical costs (for total medical costs borne by the patient, healthcare system, and third-party payer); in addition, we will refer to direct nonmedical costs as nonmedical costs.

### Study Population

The source population included pediatric and adult patients with clinician-diagnosed Lyme disease reported to public health surveillance authorities in Connecticut and Minnesota and in select counties in Maryland and New York (Appendix Table 2). Eligible patients met the national surveillance case definition for confirmed or probable disease during the study period and were referred by surveillance authorities to study personnel upon case classification (*24*). To ensure enrollment of incident cases only, we excluded the following cases: probable cases with no symptoms reported by the clinician, cases with a previous Lyme disease diagnosis within 2 calendar years of current diagnosis date, and cases with a diagnosis date >12 months before date of enrollment. Non–English-speaking patients were not enrolled because of limited resources for interpreters.

We classified eligible patients into 3 disease categories. Those with confirmed Lyme disease were divided into 2 groups: confirmed localized disease (i.e., those with erythema migrans) and confirmed disseminated disease (i.e., those with arthritis, lymphocytic meningitis, cranial neuritis or facial palsy, radiculoneuropathy, encephalomyelitis, or second- or third-degree heart block) (*24*). The third category included probable cases with symptoms reported by a clinician. To ensure enrollment of participants with a range of disease severity, we stratified recruitment by disease category and, using quota sampling, aimed to recruit approximately equal numbers of participants in each category each month. This strategy also enabled us to enroll participants as close to their diagnosis date as possible to reduce participant recall error regarding costs. Each state aimed to enroll a minimum of 50 participants per disease category; the overall minimum enrollment goal was 150 total participants per state. Recruitment and enrollment occurred during September 2014–January 2016.

### Data Collection

Participants consented to data collection for either patient costs or societal costs. Study coordinators conducted introductory telephone-based surveys with participants (or legal guardians for pediatric participants) to collect data on age, sex, annual household income, insurance coverage, and disease onset date. Patient cost data were collected at the introductory survey and then approximately monthly by using the IBM SPSS Data Collection Web Interviews survey program (IBM, https://www.ibm.com). We collected the following data on all surveys: dates for Lyme disease–related healthcare visits, clinician contact information, patient medical costs, nonmedical costs, and productivity losses. Surveys ceased when participants reported no Lyme disease–related expenses for 2 consecutive surveys or when they completed the maximum of 12 surveys.

To calculate societal medical costs, we requested billing codes (i.e., Current Procedural Terminology [CPT], 4th Edition) directly from participants’ clinicians. We requested codes from 1 month before the self-reported disease onset date to the date of the final survey. We used a date range instead of individual visit dates reported by the participant in the event participants had incorrectly reported dates. We extracted mean reimbursement for each CPT code collected for participants with private insurance from IBM MarketScan Research Databases (IBM), which include national medical claims data for privately insured persons <65 years of age and their dependents, and reimbursements for CPT codes collected for nonprivately insured participants from the Physician Fee Schedule from the Centers for Medicare and Medicaid Services (*25*). We extracted the costs of reimbursements according to state, year, and inpatient versus outpatient status (Appendix).

### Analysis

To provide an overall weighted mean and median set of reimbursements and costs, disease category sampling probabilities were estimated from disease category proportions derived from surveillance data (*4*) to approximate stratified random sampling. We then used the inverse of the sampling probabilities to weight the data for all analyses described. We excluded participants who did not complete 3 consecutive surveys from all analyses. We adjusted medical costs to 2016 US dollars by using the Consumer Price Index for medical care and the general Consumer Price Index for nonmedical costs and costs of productivity losses (*26*). We estimated the mean, median, 10th and 90th percentiles, and SDs of patient costs, societal medical costs, and total societal costs per participant. We used the Kruskal-Wallis rank-sum test to evaluate differences in cost among the 3 disease categories (confirmed localized, confirmed disseminated, probable).

To estimate the patient cost, we summed self-reported patient medical costs, nonmedical costs, and cost of productivity losses over all surveys per participant (Appendix). To calculate the societal medical costs, we summed the mean cost per CPT code collected for each participant (Appendix). Finally, we calculated the societal cost by summing the societal medical costs, patient nonmedical costs, and cost of productivity losses per participant.

We conducted multivariable linear regression analysis by using the weighted dataset to evaluate associations between the societal cost per participant and the following independent variables: disease category (confirmed localized, confirmed disseminated, probable), age group (<18, 18–45, 46–65, >65 years of age), sex (male, female), and state (Connecticut, Maryland, Minnesota, New York). We controlled for insurance status (private or nonprivate insurance), income (<$60,000 or >$60,000, which was the approximate median household income for participating states in 2015) (*27*), and study year (2014, 2015, 2016). As is typical for healthcare cost data, the distribution of societal cost was highly skewed, resulting in heteroskedasticity of the residuals in the model (*28*). Therefore, we transformed societal cost per participant by using natural logarithms and conducted sampling-weighted least squares regression (Appendix). 

We obtained research approval from institutional review boards at Centers for Disease Control and Prevention, Connecticut Department of Public Health, Maryland Department of Health, Minnesota Department of Health, New York State Department of Health, and Yale University. We conducted analyses using SAS version 9.4 (SAS Institute, https://www.sas.com) and R version 3.5.2 (29–34).

## Results

**Figure 1:**
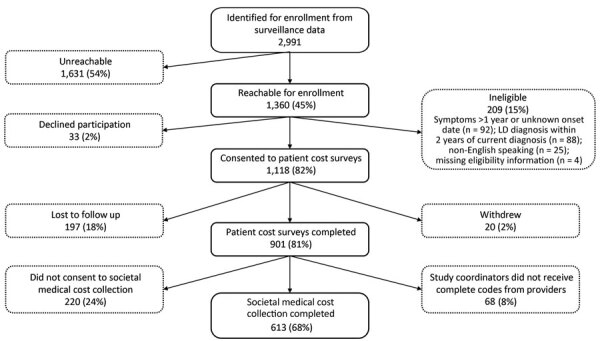
Flowchart of enrollment and completion by participants in study of economic burden of reported Lyme disease in high-incidence areas, United States, 2014–2016. LD, Lyme disease.

During the enrollment period, we identified 2,991 Lyme disease patients who were classified as having confirmed cases or probable cases with symptoms reported (Figure 1). Of the 1,360 (45%) patients we were able to contact, 1,118 (82%) consented to patient cost surveys; we included 901 (81%) participants with complete survey data in the patient cost analysis. Last, 613 (68%) of these participants also had complete societal medical cost data, and we included them in the societal cost analysis.

The study population included 402 (55%) confirmed localized, 238 (21%) confirmed disseminated, and 261 (24%) probable cases (Table 1). Overall, 36% of participants were 46–65 years of age, 57% were men, and 94% were white. Most had income >$60,000 (71%) and private health insurance (70%). Demographic distributions were similar for the 613 participants who completed both patient cost surveys and societal medical cost collection (Appendix Table 3). 

**Table 1:** Demographic characteristics of 901 participants in study of economic burden of reported Lyme disease in high-incidence areas, United States, 2014–2016

Characteristic	No. participants	Unweighted %	Weighted %
Disease category*			
Confirmed localized	402	44.6	54.5
Confirmed disseminated	238	26.4	21.2
Probable	261	29.0	24.2
Age group, y			
<18	259	28.7	28.4
18–45	145	16.1	16.1
46–65	326	36.2	36.1
>65	171	19.0	19.4
Sex			
F	385	42.7	43.1
M	516	57.3	56.9
Race			
Non-White	59	6.5	6.4
White	842	93.5	93.6
State			
Connecticut	225	25.0	23.7
Maryland	239	26.5	26.8
Minnesota	268	29.7	29.6
New York	169	18.8	20.0
Income†			
<$60,000	238	29.2	28.8
>$60,000	576	70.8	71.2
Insurance			
Private	632	70.1	70.2
Other	269	29.9	29.8

*Disease categories were derived from the surveillance case definition for Lyme disease (*24*). Those with confirmed Lyme disease were divided into 2 groups: confirmed localized disease (i.e., those with erythema migrans) and confirmed disseminated disease (i.e., those with arthritis, lymphocytic meningitis, cranial neuritis or facial palsy, radiculoneuropathy, encephalomyelitis, or 2nd or 3rd degree heart block). Those classified as probable met the probable case definition, plus had >1 symptom reported by a clinician. †Participants were not required to provide information on income; n = 814.

Participants reported a median of 2 provider visits and completed a median of 3 surveys (Table 2). Those with confirmed disseminated disease had the highest number of provider visits, reflecting the highest healthcare use, whereas those with probable disease had the highest number of surveys completed, reflecting the longest duration of costs incurred. Forty (4%) participants were still reporting symptoms and 25 (3%) were still incurring costs at survey 12.

**Table 2:** Clinician visits and duration of costs incurred, by Lyme disease category, in high-incidence areas, United States, 2014–2016

Characteristic	All	Lyme disease category		
		Confirmed localized	Confirmed disseminated	Probable
Median provider visits (range)	2 (1–47)	2 (1–25)	3 (1–45)	2 (1–47)
Median surveys* (range)	3 (1–12)	2 (1–12)	3 (1–12)	4 (1–12)

*Participants began taking surveys at study enrollment and continued at approximately 1-month intervals until they reported no Lyme disease–related expenses for 2 consecutive surveys or when they completed the maximum of 12 surveys. The following were collected on all surveys: dates for Lyme disease–related healthcare visits, clinician contact information, patient medical costs, nonmedical costs, and productivity losses.

Overall, the patient cost per participant ranged from $0.46 to $30,628. The median cost was $244 and the mean cost $1,252, reflecting a highly positively skewed distribution (Table 3). Participants with confirmed disseminated Lyme disease had the highest median cost ($358) and mean cost ($1,692), followed by those with probable disease (median $315 and mean $1,277), then participants with confirmed localized disease (median $170 and mean $1,070).

**Table 3:** Patient perspective of cost of Lyme disease per participant, by disease category, in high-incidence areas, United States, 2014–2016

Disease category	No. participants	Patient perspective, cost per participant,* 2016 US dollars					
		Median	Mean	SD	10th percentile	90th percentile	Range
All†	901	244	1,252	2,972	29	3,139	0–30,628
Confirmed localized	402	170	1,070	4,164	27	2,535	1–26,686
Confirmed disseminated	238	358	1,692	7,323	32	4,116	2–30,628
Probable	261	315	1,277	4,629	34	3,987	0–18,833

*Cost per participant according to the patient perspective represents the sum of patient medical costs, nonmedical costs, cost of productivity losses, and other related costs as reported by each participant on all surveys. †Estimates for the overall population use the sample-weighted data except the range.

**Figure 2:**
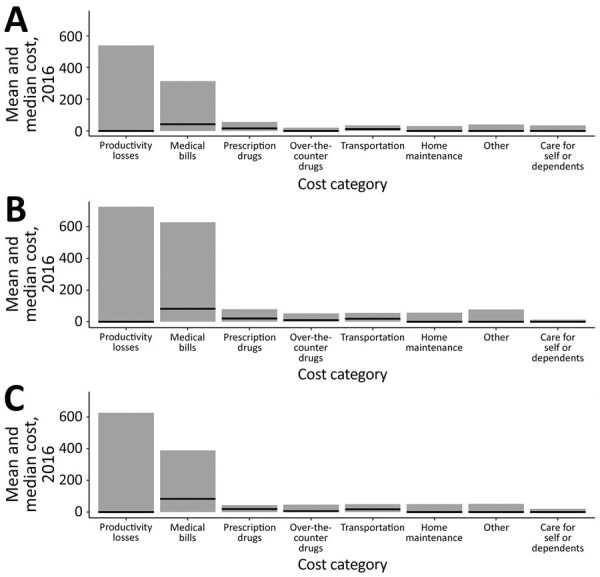
Mean and median cost per participant, by Lyme disease category and cost category of the total patient cost in high-incidence areas of the United States, 2014–2016. A) Confirmed localized disease; B) confirmed disseminated disease; C) probable disease. Black lines indicate median cost.

We calculated the median and mean cost per component of the patient cost by disease category (Figure 2; Appendix Table 4). For all disease categories, productivity losses had the highest mean cost of all cost components: $727 for those with confirmed disseminated disease, $627 for those with probable disease, and $540 for those with confirmed localized disease. However, the median cost of productivity losses for all disease categories was $0. Medical bills had the next highest cost: a median of $83 and a mean of $628 for those with confirmed disseminated disease, a median of $83 and a mean of $389 for those with probable disease, and a median of $42 and a mean of $314 for those with confirmed localized disease. All other cost components for all disease categories had median costs <$22 and mean costs <$80.

We collected 9,679 CPT codes to estimate societal medical costs. The most common codes were for office visits (17%) and routine venipuncture (6%) (Appendix Table 6). Overall, the societal medical cost per participant ranged from $50 to $121,869, for a median of $478 and mean of $1,333 (Table 4). Participants with confirmed disseminated disease had the highest median and mean societal medical cost ($696 and $2,537), followed by those with probable disease ($612 and $1,804), then confirmed localized disease ($374 and $668).

**Table 4:** Societal perspective of medical cost of Lyme disease per participant, by disease category, in high-incidence areas, United States, 2014–2016

Disease category	No. participants	Societal perspective, medical cost per participant,* 2016 US dollars					
		Median	Mean	SD	10th percentile	90th percentile	Range
All†	613	478	1,333	5,690	164	1,932	50–121,869
Confirmed localized	273	374	668	1,715	136	1,224	50–13,050
Confirmed disseminated	154	696	2,537	20,220	259	4,366	147–121,869
Probable	186	612	1,804	15,188	237	2,454	124–105,494

*Societal medical cost per participant excludes Current Procedural Terminology (CPT) codes deemed unrelated to Lyme disease as determined by a physician subject matter expert (Appendix Table 5). **†**Estimates for the overall population use the sample-weighted data except the range.

Overall, the societal cost of Lyme disease per participant ranged from $54 to $122,766; the median was $690 and the mean $2,032 (Table 5). Participants with confirmed disseminated disease had the highest median and mean societal cost ($1,081 and $3,251), followed by those with probable disease ($940 and $2,620), then confirmed localized disease ($493 and $1,307) (Appendix Table 7). Applying these per participant societal costs to estimates of the number of Lyme disease cases diagnosed each year (*6*), the aggregate cost to US society annually would be ≈$345 million using median costs and ≈$968 million using mean costs (2016 US dollars; Appendix Tables 9, 10).

**Table 5:** Societal perspective of total cost of Lyme disease per participant, by disease category, in high-incidence areas, United States, 2014–2016

Disease category	No. participants	Societal perspective, total cost per participant,* 2016 US dollars					
		Median	Mean	SD	10th percentile	90th percentile	Range
All†	613	690	2,032	6,091	203	4,201	54–122,766
Confirmed localized	273	493	1,307	3,559	154	2,678	54–18,322
Confirmed disseminated	154	1,081	3,251	20,908	297	6,238	216–122,766
Probable	186	940	2,620	15,533	316	5,021	130–105,500

*Total cost per participant according to the societal perspective includes societal medical costs, patient nonmedical costs, and cost of productivity losses. Patient medical costs were not added because they are already included in societal medical costs. †Estimates for the overall population use the sample-weighted data except the range.

In multivariable linear regression analysis, disease category, age, and state were associated with societal cost per participant (Table 6; Appendix Table 8). Costs for participants with confirmed disseminated disease were 120% higher than costs for participants with confirmed localized disease (p<0.001); participants with probable disease had costs that were 59% higher than for those with confirmed localized disease. Participants 18–45 and 46–65 years of age had costs that were 96% and 108% higher, respectively, than those <18 years of age (p<0.001); however, those >65 years of age did not have significantly different costs. Minnesota residents had 75% higher costs than did Connecticut residents, but Maryland and New York residents did not have significantly different costs from those for Connecticut residents.

**Table 6:** Impact of disease category, age group, sex, and state on total societal cost of Lyme disease per participant, United States, 2014–2016 (n = 613)*

Variable	% Difference	Total cost difference, 2016 US dollars (95% CI)
Baseline cost†	NA	305 (206–451)
Lyme disease category		
Confirmed, localized	Referent	Referent
Confirmed, disseminated	120	367 (188–545)
Probable	59	181 (71–291)
Age group, y		
<18	Referent	Referent
18–45	96	293 (107–479)
46–65	108	331 (175–486)
>65	27	84 (−28 to 195)
Sex		
F	Referent	Referent
M	11	35 (−26 to 95)
State		
Connecticut	Referent	Referent
Maryland	0	0 (−76 to 76)
Minnesota	75	229 (114–345)
New York	−6	−19 (−119 to 82)

*Results from sample-weighted multivariable linear regression analysis. The model includes independent variables of interest (i.e., disease category, age group, sex, and state), while controlling for insurance status, income, and study year (Appendix). Adjusted R^2^ = 0.19. †Baseline cost represents a patient with confirmed localized Lyme disease, female, <18 years of age, residing in Connecticut, without private insurance, with income <$60,000, in the study year of 2014.

## Discussion

In this study, persons with confirmed or probable Lyme disease had an average patient cost of ≈$1,200 (median cost ≈$240) and an average societal cost of ≈$2,000 (median cost ≈$700). In stratified analyses by disease category, those with confirmed disseminated or probable disease had double or more the societal cost per participant than those with confirmed localized disease, highlighting the importance of early and accurate diagnosis. Having disseminated or probable disease, being 18–65 years of age, and residing in Minnesota had the greatest effects on the societal cost of Lyme disease. Although median societal costs were typically <$1,000 for all disease categories, mean costs were substantially higher, indicating that most patients have low costs, but some experience very high costs related to this disease. Similarly, the low median number of provider visits and hours of lost productivity suggest that Lyme disease illness is manageable for most patients, but for a minority, it can be highly disruptive. Approximately 476,000 cases of Lyme disease are diagnosed each year in the United States, so the aggregate cost to society annually could be $345–968 million (2016 US dollars). This substantial economic burden underscores the need for effective prevention methods, such as a vaccine.

Classification of a reported case as probable means a clinician has diagnosed Lyme disease in a patient and laboratory evidence of infection exists. However, any reported symptoms are typically nonspecific and do not meet clinical criteria for a confirmed case (*24*). Further, laboratory evidence of infection includes single-tier IgG immunoblot seropositivity, which might indicate past, rather than current, infection. As such, the increased costs for probable cases might result from higher healthcare use for disease unrelated to Lyme disease, which highlights the need for improvements in Lyme disease diagnosis and clinician education.

In a geographically limited study of Lyme disease patients residing on the eastern shore of Maryland during 1998–2001, Zhang et al. (*9*) reported mean total costs of $3,494 and median total costs of $500 (2016 US dollars) per patient attributable to this disease. However, their case definition differed from ours in its inclusion of patients with early, late, and suspected disease, as well as those with tick bite and other related complaints, as identified using diagnosis codes in medical records. Therefore, these figures might not be directly comparable to our mean and median societal costs ($2,032 and $690). Zhang et al. reported mean and median total costs of $2,275 and $689 (2016 US dollars) for participants with clinically defined early disease, which are higher than what we found for confirmed localized disease (mean $1,307 and median $493). However, in regression analyses, Zhang et al. found that disease category and age, but not sex, were significantly associated with societal medical costs, similar to our findings for societal cost. In another US study using nationwide commercial insurance claims data to compare cases with matched controls during 2006–2010, Adrion et al. (*8*) estimated an increase of $3,009 (2016 US dollars) in societal medical costs attributable to Lyme disease over a 12-month period. That cost is higher than our overall mean societal medical cost ($1,333), likely because of study population differences, but is similar to that found for our participants with confirmed disseminated disease ($2,537). In a recent study in the Netherlands, Van den Wijngaard et al. (*17*) used a societal perspective to estimate a total cost of $137 for patients with erythema migrans only and $6,398 (2016 US dollars) for those with disseminated Lyme borreliosis. These costs are lower than those for our societal results for confirmed localized disease ($1,307) and higher than those for our societal results for confirmed disseminated disease ($3,251). These cost differences might result from different healthcare financing systems in the United States versus Europe or from variations in clinical manifestations resulting from infection with different *B. burgdorferi* sensu lato strains (*15*–*17*).

Our study adds to the scarce literature on the economic burden of Lyme disease and provides a comprehensive estimate of its costs to both the patient and society. In addition, our prospective collection of all patient costs, including nonmedical costs and productivity losses, enables more accurate and more comprehensive cost estimates compared with previous studies in the United States. Further, these results provide estimates of the cost savings per case averted, which can be used in cost-benefit analyses of prevention interventions, such as a potential vaccine.

The first limitation of our study is that our estimates might be affected by recall error, either by participants or providers, although we attempted to mitigate such error by enrolling patients as close to disease onset as possible, by surveying them monthly to capture ongoing costs, and by requesting codes from providers for a date range instead of for individual visits. However, by requesting codes over a date range, some billing codes unrelated to Lyme disease (e.g., for other comorbidities) might have been included despite our excluding codes definitively unrelated to Lyme disease, potentially leading to overestimates. Information bias might have occurred in our measure of association between disease category and cost because those with milder disease might be more likely to forget some costs than those with more severe disease, with a potential bias away from null. In addition, although the use of quota sampling to recruit reported cases was necessary to enroll patients near disease onset, this nonprobability sampling method limits our ability to meet assumptions for calculating sampling error. Use of surveillance data to weight responses by disease category was intended to ensure representativeness by disease category. Nevertheless, in surveillance data, confirmed localized cases are likely underreported, resulting in confirmed disseminated cases representing an artificially large proportion of cases; therefore, our overall cost might be overestimated (*35*,*36*). Finally, this study did not include costs related to deaths from Lyme disease, because no enrolled participants died. Although very rare, death from Lyme carditis has been reported (*2*,*3*), and associated productivity losses would greatly increase cost estimates.

Our results reflect the costs of diagnosed cases meeting the Lyme disease surveillance case definition in high-incidence states (*24*); as such, these costs likely reflect that of actual infections. However, we were not able to evaluate whether our estimates accurately represent the cost of diagnosed but unreported Lyme disease, cases that reflect some proportion of overdiagnosis (*6*). Further, our results might not reflect costs in states with emerging or low incidence of Lyme disease. Therefore, our results for extrapolation of costs to an estimated ≈476,000 diagnosed cases nationally per year should be interpreted with caution. Last, our results do not include costs for suspected Lyme disease (e.g., consultation and prophylactic treatment for tick bite, negative diagnostic tests), undiagnosed disease, or nonacute disease (e.g., patients experiencing long-term symptoms). These costs would further increase the total economic burden attributable to Lyme disease. Future efforts should include cost-effectiveness analyses of current and future prevention methods, such as a vaccine, in addition to economic evaluations of unreported, suspected, and nonacute disease.

In conclusion, Lyme disease represents a substantial economic burden to individual patients and US society. The aggregate cost of diagnosed Lyme disease could be nearly $1 billion annually, not including suspected, undiagnosed, or nonacute cases. These findings emphasize the importance of early and accurate diagnosis to reduce both illness and its associated personal and societal costs. 

Appendix Additional information about economic burden of reported Lyme disease in high-incidence areas, United States, 2014–2016
