# Supplementary material for: Economic Burden of Reported Lyme Disease in High-Incidence Areas, United States, 2014–2016
Source: Emerg Infect Dis. 2022 Jun;28(6):1170–9. doi: 10.3201/eid2806.211335 (PMC9155891; doi:10.3201/eid2806.211335)
Supplement: Appendix — Additional information about economic burden of reported Lyme disease in high-incidence areas, United States, 2014–2016 [file 21-1335-Techapp-s1.pdf]

Article DOI: https://doi.org/10.3201/eid2806.211335

Economic Burden of Reported Lyme Disease in High-Incidence Areas, United States, 2014–2016

Appendix

Section 1. Detailed Description of Cost Calculations for Total Patient Cost (Patient Perspective) and Societal Medical Costs (Societal Perspective)

To estimate the total patient cost, we summed self-reported medical costs, nonmedical costs, cost of productivity losses, and other related costs over all surveys. For nonmedical costs related to travel for clinician, pharmacy, or laboratory visits, the self-reported roundtrip mileage per visit was multiplied by the standard mileage rate from the Internal Revenue Service for the respective year (*1*). To calculate cost estimates for productivity losses, self-reported hours missed from work for adult participants or parents of pediatric participants were multiplied by the hourly earnings by age and sex derived from Grosse et al. (*2*), which uses the human capital approach to estimate annual market and non-market productivity from the US Census Bureau's American Community Survey and American Time Use Survey.

We calculated the societal medical cost per participant (regardless of who pays) by summing the mean cost per CPT code collected for each participant. The codes represented clinician visits, consultation and related in-office procedures, diagnostic testing, therapy, hospitalization, emergency department (ED) visits, or other procedures or relevant costs. Mean cost for each CPT code collected for participants with private insurance was extracted from IBM MarketScan Research Databases, which include national medical claims data for privately insured persons up to age 65 and their dependents. Costs for CPT codes collected for non-privately insured participants were extracted from the Physician Fee Schedule National Payment Amount File from the Centers for Medicare and Medicaid Services (CMS) (*3*). Both MarketScan and CMS costs reflect reimbursements made for charges for medical procedures and services and include the amount paid by the insurer as well as the beneficiary (such as deductibles, copays,

and coinsurance). We did not collect billing codes from pharmacies or laboratories. Therefore, we extracted the mean cost of the recommended antibiotics for Lyme disease (LD) by state and study year from MarketScan drug cost data and added this cost to each participant's total societal medical cost (*4–8*). Because laboratory evidence of infection is required to meet criteria for confirmed disseminated or probable disease (*9*), we added the cost of the recommended two-tiered LD diagnostic testing to the total societal medical cost for all participants in these disease categories who did not already have these CPT codes documented. We excluded from analysis participants for whom CPT code collection was incomplete due to provider nonresponse to code collection requests. We also excluded individual CPT codes deemed unrelated to LD, per consultation with an infectious disease physician, that were collected coincidentally from providers (Appendix Table 5).

Equation 1. Multivariable Linear Regression Model Equation

We used a multivariable linear regression model to estimate the relative impact of our independent variables of interest on the total societal cost of LD per participant. As is typical for healthcare cost data, the distribution of total cost was highly skewed, resulting in heteroskedasticity of the residuals in the model (*10*). Therefore, we transformed total societal cost per participant using natural logarithms and conducted sampling-weighted least squares regression. The basic equation is as follows:

$$\log(y_i) = \beta_0 + \beta_i x_i + \varepsilon_i$$

where Y_i, is the dependent variable, the total societal cost of LD for patient i; X_i is a vector of covariates; and ε_i is a mean-zero random error. The equation is written as follows for our specific vector of covariates (i.e., independent variables of interest and potential confounders):

$$\log(y_i) = \beta_0 + \beta_1\text{Disease category} + \beta_2\text{Age group} + \beta_3\text{Sex} + \beta_4\text{State} +$$

$$\beta_5\text{Insurance status} + \beta_6\text{Income} + \beta_7\text{Study year} + \varepsilon_i$$

Baseline costs came from the intercept term, β_0, which represents a patient with confirmed localized Lyme disease, female, aged <18 years, with residence in CT, without private insurance, with income <$60,000, and study year of 2014. Resulting β coefficients were back

transformed by exponentiation, interpreted as the multiplicative difference in the geometric mean of the total cost of Lyme disease for a 1-unit difference in the independent variable of interest after adjusting for confounders. For interpretability, we calculated the percent difference in cost from baseline for each level of each independent variable, excluding reference (i.e., baseline) levels (Percent difference = (Exp(coefficient) – 1) * 100). These additional costs were added or subtracted to the baseline costs for each independent variable of interest (Table 6, LINK; Appendix Table 8).

Section 2. Description of Calculations for Extrapolation of Total Societal LD Cost Per Participant to Annual, Aggregate Total LD Cost to U.S. Society

Previous research has demonstrated that LD surveillance case numbers are likely 8–12-fold underreported (*11*,*12*), with a recent study estimating 476,000 diagnosed cases per year (*13*). The table below shows total cases by confirmed localized, confirmed disseminated, and probable disease per the proportions found in surveillance data. Case numbers by disease category are multiplied by the mean and median total cost per participant estimated from this study to get the aggregate cost per disease category (Appendix Table 9). When summed, the mean total cost of LD in the U.S. annually is approximately $968,444,834, while the median is $345,164,936 (2016 USD, Appendix Table 10). In 2020 USD, the mean cost is $1,039,260,297 using the Consumer Price Index (CPI) for all consumers and $1,078,657,584 when using the CPI for medical care (*14*), with median costs at $370,404,385 and $384,446,034, respectively (Appendix Table 10).

References

1. Standard Mileage Rates IRS. 2019 [cited 2019 7/22/2019]; Available from: https://www.irs.gov/tax-professionals/standard-mileage-rates

2. Grosse SD, Krueger KV, Pike J. Estimated annual and lifetime labor productivity in the United States, 2016: implications for economic evaluations. J Med Econ. 2019;22:501–8. PubMed https://doi.org/10.1080/13696998.2018.1542520

3. Centers for Medicare and Medicaid. PFS National Payment Amount File. 2019 [cited 2019 Nov 6]. https://www.cms.gov/Medicare/Medicare-Fee-for-Service-Payment/PhysicianFeeSched/PFS-National-Payment-Amount-File.html

4. Hu LT. Lyme Disease. Ann Intern Med. 2016;165:677. PubMed https://doi.org/10.7326/L16-0409

5. Kowalski TJ, Tata S, Berth W, Mathiason MA, Agger WA. Antibiotic treatment duration and long-term outcomes of patients with early Lyme disease from a Lyme disease-hyperendemic area. Clin Infect Dis. 2010;50:512–20. PubMed https://doi.org/10.1086/649920

6. Sanchez E, Vannier E, Wormser GP, Hu LT. Diagnosis, Treatment, and Prevention of Lyme Disease, Human Granulocytic Anaplasmosis, and Babesiosis: A Review. JAMA. 2016;315:1767–77. PubMed https://doi.org/10.1001/jama.2016.2884

7. Stupica D, Lusa L, Ruzić-Sabljić E, Cerar T, Strle F. Treatment of erythema migrans with doxycycline for 10 days versus 15 days. Clin Infect Dis. 2012;55:343–50. PubMed https://doi.org/10.1093/cid/cis402

8. Centers for Disease Control and Prevention. Lyme disease treatment. 2020 [cited 2020 Feb 13]. https://www.cdc.gov/lyme/treatment/index.html

9. Centers for Disease Control and Prevention. Lyme Disease (Borrelia burgdorferi) 2008 Case Definition. 2008 [cited 2019 Oct 18]. https://wwwn.cdc.gov/nndss/conditions/lyme-disease/case-definition/2008/

10. Mihaylova B, Briggs A, O'Hagan A, Thompson SG. Review of statistical methods for analysing healthcare resources and costs. Health Econ. 2011;20:897–916. PubMed https://doi.org/10.1002/hec.1653

11. Nelson CA, Saha S, Kugeler KJ, Delorey MJ, Shankar MB, Hinckley AF, et al. Incidence of clinician-diagnosed Lyme disease, United States, 2005–2010. Emerg Infect Dis. 2015;21:1625–31. PubMed https://doi.org/10.3201/eid2109.150417

12. Hinckley AF, Connally NP, Meek JI, Johnson BJ, Kemperman MM, Feldman KA, et al. Lyme disease testing by large commercial laboratories in the United States. Clin Infect Dis. 2014;59:676–81. PubMed https://doi.org/10.1093/cid/ciu397

13. Kugeler KJ, Schwartz AM, Delorey MJ, Mead PS, Hinckley AF. Estimating the frequency of Lyme Disease diagnoses, United States, 2010–2018. Emerg Infect Dis. 2021;27:616–9. PubMed https://doi.org/10.3201/eid2702.202731

14. U.S. Bureau of Labor Statistics. Databases, Tables & Calculators by Subject. 2020 [cited 2020 Sep 30]. https://data.bls.gov/pdq/SurveyOutputServlet

15. Meltzer MI. Introduction to health economics for physicians. Lancet. 2001;358:993–8. PubMed https://doi.org/10.1016/S0140-6736(01)06107-4

Appendix Table 1. Cost categories and inclusion of costs dependent on perspective for economic analysis*

Cost categories	Examples of costs	Perspective†	
		Patient	Society‡
Direct medical			
	Healthcare personnel time	Included	Included
	Drugs or other therapy	Included	Included
	Laboratory tests	Included	Included
	Medical devices, supplies, equipment	Not included	Included
Direct nonmedical			
	Administrative resources	Not included	Included
	Healthcare facility	Not included	Included
	Utilities	Not included	Included
	Patient’s travel cost	Included	Included
	Hired caregiving	Included	Included
	Hired household help	Included	Included
Indirect			
	Time off work for healthcare visits	Included	Included
	Time off work due to symptoms	Included	Included

*Source: adapted from Meltzer MI (*15*).
†Economic analyses are usually conducted from the following perspectives: patient, clinician, hospital, third-party payer, and/or society.
‡The societal perspective includes all costs related to an illness, no matter who pays, and is typically the sum of costs paid by the patient, clinician, hospital, and third-party payer.

Appendix Table 2. Participating counties from Maryland and New York

State	County
Maryland	Anne Arundel
	Baltimore
	Calvert
	Carroll
	Cecil
	Frederick
	Harford
	Howard
	Montgomery
New York	Albany
	Rensselaer
	Columbia
	Greene
	Saratoga
	Schoharie
	Schnectady
	Washington
	Fulton
	Montgomery
	Ostego
	Delaware
	Dutchess
	Ulster

Appendix Table 3. Characteristics of the subset of participants with complete patient cost and societal medical cost data, n = 613

Characteristic	Category	No.	Unweighted %	Weighted %
Disease category	Confirmed localized	273	44.5	54.4
	Confirmed disseminated	154	25.1	20.2
	Probable	186	30.3	25.4
Age group, y	<18	173	28.2	27.7
	18–45	96	15.7	15.7
	46–65	228	37.2	37
	>65	116	18.9	19.5
Sex	Female	262	42.7	43.0
	Male	351	57.3	57.0
Race	Non-White	45	7.3	7.1
	White	568	92.7	92.9
State	CT	128	20.9	19.3
	MD	203	33.1	33.6
	MN	191	31.2	31.1
	NY	91	14.8	15.9
Income*	≤$60,000	160	28.7	28.4
	>$60,000	398	71.3	71.6
Insurance	Private	438	28.5	28.5
	Other	175	71.5	71.5
Median clinician visits (range)		2 (1–33)	NA	NA
Median surveys (range)		3 (1–12)	NA	NA

*Participants were not required to provide information on income; n = 558.

Appendix Table 4. Mean and median patient Lyme disease costs per participant, by disease and cost category, 2016 US dollars*

	Confirmed localized disease		Confirmed disseminated disease		Probable disease	
Cost category	Median (95% CI)	Mean (95% CI)	Median (95% CI)	Mean (95% CI)	Median (95% CI)	Mean (95% CI)
Productivity losses	0 (0–0)	540 (368–712)	0 (0–0)	727 (359–1095)	0 (0–0)	627 (429–824)
Medical bills	42 (31–52)	314 (201–426)	83 (61–145)	628 (408–848)	83 (62–99)	389 (264–514)
Prescription medicine	16 (11–21)	56 (32–80)	21 (17–21)	79 (54–105)	19 (16–21)	44 (34–54)
Over-the-counter medicine	0 (0–4)	20 (13–27)	10 (7–10)	53 (28–78)	5 (2–8)	47 (21–72)
Transportation	11 (10–12)	34 (16–52)	20 (17–23)	55 (37–74)	18 (14–20)	49 (30–69)
Home maintenance	0 (0–0)	31 (7–55)	0 (0–0)	58 (12–103)	0 (0–0)	50 (4–97)
Other	0 (0–0)	40 (23–58)	0 (0–0)	78 (37–118)	0 (0–0)	51 (27–75)
Care for self/dependents	0 (0–0)	35 (0–91)	0 (0–0)	14 (0–28)	0 (0–0)	20 (0–42)

Appendix Table 5. List of CPT codes removed from analyses, code description, frequency, mean cost per code (n = 30)*

CPT code	Code description	N	Mean cost (2016 USD)
11406	Removal of growth (4.0 cm) of the trunk, arms, or legs	1	1152
11606	Removal of malignant growth (over 4.0 cm) of the trunk, arms, or legs	1	1240
36475	Destruction of insufficient vein of arm or leg, accessed through the skin	1	3021
37609	Tying or biopsy of temporal artery (side of skull)	1	1844
42821	Removal of tonsils and adenoid glands patient age 12 or over	1	1946
42830	Removal of adenoids patient younger than age 12	1	1854
43238	Ultrasound guided needle aspiration or biopsies of esophagus using an endoscope	1	1664
45378	Diagnostic examination of large bowel using an endoscope	4	752
45380	Biopsy of large bowel using an endoscope	1	1131
45385	Removal of polyps or growths of large bowel using an endoscope	2	2911
47562	Removal of gallbladder using an endoscope	1	4062
55700	Biopsy of prostate gland	1	679
59510	Cesarean delivery with pre- and post-delivery care	1	2893
62311	Injections of substances into lower or sacral spine	3	565
64490	Injections of upper or middle spine facet joint using imaging guidance	1	788
64491	Injections of upper or middle spine facet joint using imaging guidance	1	531
64492	Injections of upper or middle spine facet joint using imaging guidance	1	502
64635	Destruction of lower or sacral spinal facet joint nerves using imaging guidance	1	1155
64636	Destruction of lower or sacral spinal facet joint nerves with imaging guidance	1	801
66984	Removal of cataract with insertion of lens	3	2415
69436	Incision of eardrum with insertion of eardrum tube under general anesthesia	1	1802
78582	Lung ventilation and perfusion imaging	1	598
92928	Catheter insertion of stents in major coronary artery or branch, accessed through the skin	1	3530

CPT code	Code description	N	Mean cost (2016 USD)
95810	Sleep monitoring of patient (6 y or older) in sleep lab	1	1535
C1725	Catheter, transluminal angioplasty, non-laser (may include guidance, infusion/perfusion capability)	2	2182
C1874	Stent, coated/covered, with delivery system	1	5411
C9600	Percutaneous transcatheter placement of drug eluting intracoronary stent(s), with coronary angioplasty when performed; single major coronary artery or branch	1	12616
G0202	Screening mammography digital	14	490
G0206	Diagnostic mammography digital	3	523
J0133	Injection, acyclovir, 5 mg	1	824

*An infectious disease physician with subject matter expertise in Lyme disease deemed these CPT codes to be unrelated to Lyme disease; 54 instances with these 30 codes recorded were deleted from the dataset before all analyses.

Appendix Table 6. Twenty-five most frequently reported CPT codes, code description, and summary statistics

CPT code	Code description	N	Proportion	MarketScan mean, median reimbursement (USD 2016)	CMS mean, median reimbursement (USD 2016)
99213	Established office visit	970	0.1	100.55, 85.14	75.04, 74.70
99214	Established patient office or other outpatient, visit typically 25 min	707	0.07	144.43,120.56	112.68,115.18
36415	Routine venipuncture	579	0.06	8.40, 3.89	3.00, 3.00
Q9967	LOCM 300–399mg/ml iodine,1ml	344	0.04	336.87,165.17	152.19,165.17
86617	Confirmation test for antibody to Borrelia burgdorferi (Lyme disease bacteria)	308	0.03	64.58, 32.82	28.49, 28.49
85025	Complete blood cell count (red cells, leukocyte, platelets), automated test	287	0.03	26.57, 18.03	14.30, 14.30
86618	Analysis for antibody Borrelia burgdorferi (Lyme disease bacteria)	273	0.03	42.44, 33.62	31.32, 31.32
J3490	Unclassified drugs	180	0.02	489.57, 28.90	37.95, 28.90
97110	Therapeutic exercises	166	0.02	64.88, 49.72	33.17, 32.36
J1642	Injection, heparin sodium, (heparin lock flush), per 10 units	130	0.01	248.86, 24.21	24.21, 24.21
80053	Blood test, comprehensive group of blood chemicals	105	0.01	40.06, 14.87	19.43, 19.43
97112	Neuromuscular reeducation	102	0.01	44.74, 25.22	34.03, 33.71
86140	Measurement C-reactive protein for detection of infection or inflammation	96	0.01	21.47, 9.49	9.52, 9.52
98940	Chiropractic manipulative treatment, 1–2 spinal regions	88	0.01	38.20, 28.00	29.52, 29.43
80048	Blood test, basic group of blood chemicals	87	0.01	33.53, 18.90	15.55, 15.55
J0696	Injection, ceftriaxone sodium, per 250 mg	85	0.01	165.44, 32.76	44.70, 32.76
A9585	Injection, gadobutrol, 0.1 ml	83	0.01	228.98, 90.00	90.01, 90.00
99212	Established patient office or other outpatient visit, typically 10 min	82	0.01	68.39, 51.74	45.35, 45.11
90471	Administration of 1 vaccine	78	0.01	26.87, 24.47	26.56, 25.52
99203	New patient office or other outpatient visit, typically 30 min	77	0.01	138.31,109.00	112.28,116.07
85652	Red blood cell erythrocyte sedimentation rate, to detect inflammation	76	0.01	10.53, 4.73	4.97, 4.97
93000	Routine EKG using at least 12 leads including interpretation and report	75	0.01	47.41, 38.79	17.95, 18.34
87880	Strep test (Streptococcus, group A)	70	0.01	20.26, 17.31	22.05, 22.05
99284	Emergency department visit, problem of high severity	70	0.01	532.25,411.12	119.94,120.87
85027	Complete blood cell count (red cells, leukocyte, platelets), automated test	65	0.01	23.06, 13.97	11.90, 11.90

Appendix Table 7. Societal Perspective: total cost of Lyme disease per participant, by demographic characteristic, n = 613

Characteristic	Category	N	Mean (2016 US dollars)	Median (2016 US dollars)
Disease category	Confirmed localized	273	1307	493
	Confirmed disseminated	154	3251	1081
	Probable	186	2620	940
Age group, y	<18	173	1550	503
	18–45	96	2100	960
	46–65	228	2421	1136
	>65	116	1926	521
Sex	Female	262	1417	646
	Male	351	2497	741
Race	Non-White	45	2188	774
	White	568	2021	685
State	CT	128	3307	621
	MD	203	1493	604
	MN	191	2112	1124
	NY	91	1470	434
Income*	≤$60,000	160	2159	685
	>$60,000	398	2088	696
Insurance	Private	438	2295	807
	Other	175	1376	578

*Participants were not required to provide information on income; n = 558.

Appendix Table 8. Multivariable linear regression results: factors influencing total societal cost of Lyme disease per participant (n = 613)*

Variable	Coefficient	Standard error of coefficient	Exp(coefficient)[2]	Percent difference (%)	Cost difference (2016 USD)	95% CI for cost difference (2016 USD)	P value
Baseline (Intercept)[4]	5.72	0.20	305.08	NA	NA	206.28 – 451.20	<0.001
Confirmed disseminated	0.79	0.11	2.20	120	366.58	188.12 – 545.04	<0.001
Probable	0.47	0.10	1.59	59	181.13	70.84 – 291.42	<0.001
18–45 y	0.67	0.15	1.96	96	292.99	107.11 – 478.88	<0.001
46–65 y	0.73	0.11	2.08	108	330.79	175.08 – 486.50	<0.001
>65 y	0.24	0.17	1.27	27	83.65	−27.51 – 194.81	0.15
Male	0.11	0.09	1.11	11	34.56	−25.67 – 94.8	0.24
MD	0.00	0.13	1.00	0	−0.01	−75.98 – 75.97	1.00
MN	0.56	0.13	1.75	75	229.44	113.85 – 345.03	<0.001
NY	−0.06	0.17	0.94	−6	−18.52	−118.96 – 81.91	0.72
Privately insured	0.24	0.15	1.27	27	82.53	−23.22 – 188.28	0.11
>$60,000 income	−0.06	0.12	0.94	−6	−18.52	−90.11 – 53.08	0.61
Study year 2015	−0.07	0.10	0.93	−7	−21.35	306.05 – 427.11	0.48
Study year 20156	0.50	0.50	1.65	65	197.52	−306.37 – 668.64	0.32

*The model included independent variables of interest, i.e., disease category, age group, sex, and state, while controlling for insurance status, income, and study year. Reference levels are not shown but are described in Table **6** of main article (https://wwwnc.cdc.gov/EID/article/28/6/21-1335-T5.htm). Adjusted R^2 = 0.19.

†Beta coefficients were back transformed by exponentiation, interpreted as the multiplicative difference in the geometric mean of the total cost of Lyme disease for a 1-unit difference in the independent variable of interest after adjusting for confounders.

‡Percent difference = (Exp(coefficient) – 1) * 100; this represents the percent change in cost from baseline for each level of each variable, excluding reference (i.e., baseline) levels.

§Baseline cost (i.e., e^{β_0}) represents a patient with confirmed localized Lyme disease, female, aged <18 y, with residence in CT, without private insurance, with income <$60,000, and study year of 2014.

Appendix Table 9. Inputs for extrapolation of total Lyme disease cost per participant to aggregate total Lyme disease cost to US society

Lyme disease category	Estimated proportion of total cases	Total cases	Mean total cost per participant*	Total aggregate cost using mean cost per participant*	Median total cost per participant*	Total aggregate cost using median cost per participant*
Confirmed localized	0.547	260,435	1,307	340,388,077	493	128,394,278
Confirmed disseminated	0.211	100,278	3,251	326,004,293	1,081	108,400,689
Probable	0.242	115,287	2,620	302,052,464	940	108,369,968

*2016 USD

Appendix Table 10. Estimated annual total Lyme disease cost to US society

Category	Mean	Median
Total (2016 USD)	968,444,834	345,164,936
Total (2020 USD)*	1,039,260,297	370,404,385
Total (2020 USD, medical CPI)†	1,078,657,584	384,446,034

*Converted to 2020 USD using Consumer Price Index, “All items in U.S. city average, all urban consumers, not seasonally adjusted” (CPI-U, Series ID: CUUR0000SA0). 2016 annual average CPI and 2020 first half of year average CPI used. Data extracted from https://data.bls.gov/pdq/SurveyOutputServlet on 9/30/2020.

†Converted to 2020 USD using Consumer Price Index, “Medical care in U.S. city average, all urban consumers, not seasonally adjusted” (CPI-U Medical care, Series ID: CUUR0000SAM). 2016 annual average CPI and 2020 first half of year average CPI used. Data extracted from https://data.bls.gov/pdq/SurveyOutputServlet on 9/30/2020.
